# Tooth- and Patient-Related Conditions May Influence Root Canal Treatment Indication

**DOI:** 10.1155/2021/7973356

**Published:** 2021-12-30

**Authors:** Maria Tereza Pedrosa Albuquerque, Lorena Caetano Abreu, Leticia Martim, Eliseu Aldrighi Münchow, Juliana Yuri Nagata

**Affiliations:** ^1^Department of Clinical Dentistry Endodontics, Federal University of Bahia, Av. Araújo Pinho 62, Salvador 40301-155, Brazil; ^2^Dentistry Department Endodontics, Federal University of Sergipe, Av. Gov. Marcelo Déda 300- Lagarto, São José 49400-000, Brazil; ^3^Federal University of Rio Grande do Sul/Department of Conservative Dentistry, Rua Ramiro Barcelos 2492, Porto Alegre 90035-004, Brazil

## Abstract

**Aim:**

To investigate predisposing aspects related to the indication for root canal treatment (RCT) at Dental Schools of Brazil.

**Methods:**

Data of 207 patients referred to endodontic treatment at undergraduate Dental Schools of Brazil were collected over a period of 8 months. Patient-related data included age, gender, first dental visit, tooth brushing frequency, number of remaining teeth, and previous endodontic treatment, and tooth-related information regarding number, type, and location in the mouth of teeth that need RCT, waiting time for the endodontic treatment, endodontic diagnosis, pain report, and dental crown condition was collected for each participant. Bivariate analysis (Chi-square test; *p* < 0.05) associated gender and tooth/patient-related conditions. Poisson regression analysis compared multiple endodontic need and exposure variables.

**Results:**

Patients were mainly comprised of women (65.2%), aged 18–39 years (69.6%). Previous RCT was significantly more prevalent in women when compared to men (*p*=0.005). First dental visit at later moment (after 20 years of age) demonstrated 400% greater prevalence of multiple RCT demand when compared to patients that attended the dental office at age below seven years (*p*=0.032). Most of the patients presented only one tooth needing RCT (80.2%), mainly premolars (44.9%). Interestingly, women underwent more RCT in maxillary teeth (*p*=0.016) and significantly contributed with their report to the diagnosis process when compared to men (*p*=0.014). Regarding dental crown condition, 45.4% of all teeth registered unsatisfactory restorations, which were more pronounced in female patients (*p*=0.018). Unsatisfactory restorations or dental caries had 75% and 82%, respectively, less prevalence of multiple RCT indication when compared to sound dental crown (*p*=0.001).

**Conclusions:**

RCT was undertaken more frequently in young female adults' premolars presenting unsatisfactory restorations. First dental visit, number of teeth with previous endodontic treatment, tooth group, and dental crown condition were significantly associated with the necessity of multiple endodontic treatments.

## 1. Introduction

Worldwide population has extended life expectancy in 10 years, requiring all kinds of health care, including dentistry, to achieve a better quality of life (QOL) [1]. A healthy-look dentition has been associated with positively influencing QOL of patients, so that endodontic treatment might be required to achieve a desirable oral health condition. Some less favored population in the world may not afford a root canal treatment (RCT), and the only other option to reach oral health relies on tooth extraction/loss, which may lead to an unhealthy-look dentition and consequently to social isolation, limited participation in community activities, and negative judgement toward society [2]. Under these circumstances, endodontic treatment has been considered the most required dental treatment in the public service of Brazil (21.4–68.4%) [3, 4]. This high demand burdens the available services delaying time to initiate/conclude RCT (∼71 days), inducing patients to choose tooth extraction in an attempt to eliminate pain [3–5].

Historically, increased need for RCT in a population could be connected to high risk of dental caries due to the expanded consumption of industrialized food [6–8]. Besides dental caries, reasoning about the factors related to endodontic treatment indication may be related to the presence of unsatisfactory restorations (83.5%), which was revealed as one of the main causes for RCT indication among a Swedish population, followed by dental caries (62.9%) [2, 9]. Although the cause/effect correlation between crown features and RCT is essential to guide the professional toward treatment plan, there is only one study in the literature evaluating crown features before endodontic treatment [9]. Moreover, it is important to mention the high demand for endodontic treatment around the world, reaching both developed countries such as Germany (58%) and Austria (49%), [10, 11] and undeveloped ones, including Brazil and Nigeria (21%–68%) [3, 4, 12].

Concomitantly to this high demand, new technologies and materials have been developed to avoid unnecessary tooth wear and loss, which represents a countless progress to reestablish tooth function in the mouth. Nevertheless, and despite the great advance in terms of rehabilitation materials and techniques, preventive strategies should be the central topic to recover oral health and preclude the need for RCT. Of note, endodontic treatment prevention should start from the study of a wide spectrum of patient/tooth-related features including individual dental history, daily behavior, and the application of more conservative techniques (*e.g*., pulp capping and partial/total pulpotomy) during dental caries management. The simple presence of the pulp into the pulp chamber ensures defense mechanisms to the whole tooth such as proprioception, tertiary dentin deposition, inflammatory/immunologic reaction, hydration, and nutrition, thus making the dental structure less friable and less susceptible to fractures [13].

Although oral health promotion inside communities has been done, there is still a failure connecting patient/tooth conditions to RCT need. No less important, the reciprocity connecting RCT to unsatisfactory restorations can build new perspectives for efficient preventive protocols, since few studies have investigated the association between tooth-crown condition and the indication for endodontic treatment [9, 14]. Thus, this study aimed to assess the dental crown-related conditions and patient dental history as possible influencing factors to the necessity of RCT in the northeast region of Brazil, testing the hypothesis that dental history of patients and tooth previous structure conditions do not influence the number of teeth requiring root canal treatment.

## 2. Methods

### 2.1. Ethical Considerations

This multicentric and cross-sectional study was approved by the regional Ethical Committee of Federal University of Sergipe, Brazil.

### 2.2. Participants' Recruitment

Individuals scheduled for endodontic treatment at dental clinics of two federal universities in northeast Brazil, between February and December 2019, were invited to contribute to this research by filling a patient-related form. A total of 241 patients were referred for endodontic treatment, and 207 patients matched inclusion/exclusion criteria and were invited to participate in this research. Each individual who agreed to participate in this study signed a written informed consent. 34 patients did not fill the inclusion criteria (25 were retreatment cases; 5 consisted of teeth with periodontal disease; and 4 had open-apex teeth) and were excluded.

The inclusion criteria were the following:

1. At least one tooth indicated for RCT during the selected period
2. Age above 18 years and ability to give voluntary informed consent

The exclusion criteria were the following:

1. Tooth presenting pathological periodontal mobility
2. Periodontal disease
3. Immature teeth

### 2.3. Data Collection

Each participant was interviewed at the time of clinical care and had the teeth conditions evaluated by two trained undergraduate students under the supervision of the responsible researcher at the beginning of the endodontic treatment. Once the information was collected, it was transcribed to a printed form covering patient- and tooth-specific characteristics. Patient-specific features included gender, age, first dental visit, daily tooth brushing routine, number of remaining teeth, and previous endodontic treatment, as well as tooth-specific characteristics such as number of teeth indicated for RCT, tooth position, tooth group, waiting time to initiate treatment, pain reported during anamnesis (e.g., none, spontaneous, shooting, aggravated by cold/hot beverages or biting/chewing), history of pain before RCT, pulp and periapical diagnosis, dental crown condition, type of restoration, number of restored surfaces, tooth substance loss, indication to periodontal surgery, and type of restoration indicated after completion of endodontic treatment.

**Figure 1:**
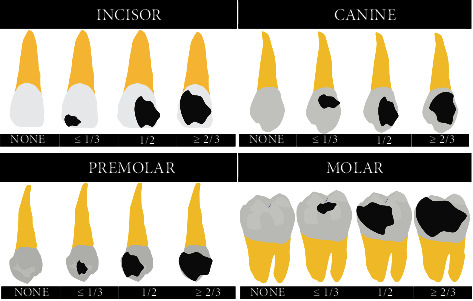
Representative images of tooth crown structure aspects before endodontic treatment according to tooth group.

Pulp diagnosis was defined using patient's pain report and sensitivity to cold test, being classified in four categories: normal pulp, necrosis, and asymptomatic and symptomatic irreversible pulpitis. Occurrence of spontaneous pain, lingering thermal sensitivity to cold test, and tenderness to percussion were classified as symptomatic irreversible pulpitis, while teeth with pulp exposure without severe spontaneous pain nor lingering thermal sensitivity, and mild tenderness to percussion, without any radiographic apical lesion, were classified as asymptomatic irreversible pulpitis. Pulp necrosis was stated in the absence of pain to cold sensitivity test. The periapical index (PAI) was used to score cases with periapical rarefaction and to define periapical condition during diagnosis [15] and tooth substance loss was classified according to Figure 1. Data was identified by an anonymous number and written in an Excel data sheet (Microsoft Corp., Redmond, WA, USA).

### 2.4. Statistical Analyses

Data were entered into an electronic spreadsheet and analyzed using the software SPSS Statistics version 22 (IBM). The prevalence of the outcomes was assessed using descriptive analysis, and the association of gender with independent variables was evaluated in bivariate way by Chi-square test, considering the significance level of *p* < 0.05. Poisson regression was used to assess the association of multiple needs for RCT (i.e., minimum of two teeth requiring endodontic treatment per each patient) with exposure variables, adjusting by possible confounders [16]. Demographic variables were placed on more distal positions, followed by dental history and behavioral characteristics. Variables related to endodontic and clinical conditions of teeth needing RCT were positioned in the most proximal part of the model. Prevalence ratios and 95% confidence intervals were estimated from the models.

## 3. Results

Data on the participants and bivariate analysis of the investigated variables are presented in Table 1. 207 patients were included in this study, of which 65.2% were female with mean age ranging from 18 to 39 years (69.6%). Most of them had their first dental visit after 7 years of age (78.7%) and reported brushing their teeth at least three times a day (58.5%). Almost 74% of the sample had 23 or more remaining teeth, with more than half (53.2%) and 23.2% of the participants exhibiting one and two previous endodontic treatments, respectively. This former RCT was significantly more prevalent in women when compared to men (*p*=0.005). Most of the patients had only one tooth indicated for RCT (80.2%), mainly maxillary teeth (73.9%) and particularly involving premolars (44.9%), followed by incisors or canines (42.5%). These maxillary teeth requiring endodontic treatment were significantly more common among women (*p*=0.016). Regarding the waiting time to initiate the treatment, most patients reported a period shorter than 15 days (68.6%). Teeth undertaking RCT were mostly diagnosed as pulp necrosis (62.8%), without periapical lesion (44.4%).

Patient's report and clinical evaluation were commonly registered in 61.4% of the cases, but females' report contributed more to the diagnosis process when compared to males (*p*=0.014). Most of the patients did not mention pain during diagnosis (59.4%), although women significantly experienced more pain than men (*p*=0.015). The majority of the sample did not feel pain at the time of the diagnosis (42.5%), but periods longer than 30 days until starting treatment demonstrated a greater symptomatic condition when compared to shorter periods. Regarding dental crown condition, 45.4% of all teeth registered unsatisfactory restorations, followed by the presence of dental caries (34.3%), wear caused by bruxism (9.7%), or satisfactory restorations (5.8%). Only few patients presented sound teeth (4.8%). Unsatisfactory restorations were more pronounced in female patients (*p*=0.018). Interestingly, the main type of restorative material identified in teeth needing RCT was temporary materials (48.2%), followed by composite resin (37.5%), amalgam (12.5%), and porcelain/ceramic (1.8%). The number of restored surfaces involved predominantly less than two surfaces (∼76%), and the extent of tooth substance loss was commonly less than 2/3 of the tooth crown (62.3%). Concerning the need for crown lengthening surgery prior to RCT and restoration plan, most of cases (84.5%) showed optimal periodontal measures, excluding any surgical procedure. Last, direct restorations were more required after RCT (78.3%) than indirect ones.

Table 2 shows the results of Poisson regression analysis which associated the collected variables with multiple indications of RCT (i.e., minimum of two teeth requiring endodontic treatment per each patient). The need of multiple RCT assembled two or more teeth requiring endodontic treatment irrespective of their group, mouth position, and diagnosis. After adjustments, four variables displayed significant association with multiple RCT indication: age of the first dental visit, number of teeth with previous endodontic treatment, tooth group, and dental crown condition. Attending the first dental visit after completing 20 years of age demonstrated 400% more prevalence of multiple RCT need when compared to patients that attended dental office aged below seven years (*p*=0.032). The presence of one tooth with previous endodontic treatment resulted in 67% less indication of multiple RCT as compared with patients without any endodontic experience (*p*=0.018). Patients needing RCT of premolars demonstrated 64% less need for multiple RCT when compared to patients having RCT indication in incisors or canines (*p*=0.020). The presence of unsatisfactory restorations or dental caries in teeth needing RCT exhibited 75% and 82%, respectively, less prevalence of need for multiple RCT when compared to sound teeth (*p*=0.001). Last, patients presenting teeth restored with amalgam showed 289% more prevalence of multiple RCT indication than patients possessing teeth with temporary restorations, despite the lack of statistical significance (*p*=0.093).

## 4. Discussion

The results of the study rejected the hypothesis since patient- and tooth-related features influenced the number of root canal treatment indications in Dental Schools of low-income region of Brazil. Prevention of endodontic pathologies through proper diagnosis and prophylactic interventions such as early caries diagnosis and adequate management, repair of unsatisfactory restorations, and occlusal adjustments may represent a decisive step during health promotion, perhaps minimizing the need of endodontic treatment. Notably, once endodontic instrumentation performed during RCT may increase the risk of dental fracture after completion of treatment, a good preventive practice should be the focus in order to anticipate strategies for preventing the need of RCT [17]. It is also recently accepted that complete pulp tissue removal may predispose the patient to recurrent caries [18]. Thus, this study described the main features related to endodontic treatment indication, as well as the dental profile of patients in a low-income population receiving dental care in public Dental Schools in northeast region of Brazil.

The sample was mainly composed of young women (65.2%), aged below 40 years (69.6%), who were significantly more associated with previous RCT compared to men (*p*=0.005). These numbers agree with previous studies conducted in Brazil, Germany, and Nigeria (62.5%, 52%, and 64.9%, respectively) [12, 14, 19]. On the other hand, our findings differ from a Swedish population record, which quantified higher prevalence of older individuals (48.3 years old) needing RCT and regardless of gender predilection [9]. This higher prevalence of RCT in young women may be related to occupational status of female in the last years, which have been strongly engaged in outside work (68.2%), so they are experiencing a stressful lifestyle, leading to poor health behaviors [20]. Also, individuals under stressful conditions may present decreased salivary flow, [21] which in turn contributes to the development of dental caries. This assignment was previously reported in Japan, where a higher risk of untreated dental caries between women who worked in stressful workplaces was found as compared to men and homeworkers [20]. However, it may also indicate that females are more concerned with their oral health, thus looking for treatment before caries progression to a level in which tooth extraction is the ultimate treatment option [22].

In this study, most of the participants attended a first dental visit after the age of seven years (78.7%) and reported brushing their teeth three times a day (58.5%). These findings disagree with International Recommendations by the American Pediatric Dentistry Academy, which advocate the first dental visit of a child to occur within six months from the eruption of the first primary tooth [23]. Alarming data were also observed in south of Brazil, where only 14/639 children reported the first visit to a dental office within the first twelve months of life, as well as in a research conducted in Poland in which first dental visits were mostly reported to occur after three years of age (38.6%) [24]. Notably, once the first dental visit takes place at later stages, visible caries and the related complications might feature the most common reason for this first dental appointment (59.86%–75.9%), confiscating the opportunity for the child to first adapt to the dentist before the need of any invasive dental treatment [25, 26]. In our study, first dental visit after completing 20 years of age reflected in a 400% greater prevalence of multiple endodontic indications when compared to patients visiting the dental office earlier (e.g., before the age of seven). Late professional guidance favors the evolution of caries in deciduous teeth and consequently increases the risk of permanent caries development, which in turn may promote pulp and periapical injuries [27, 28]. To our knowledge, this is the first study associating the time of initial dental office visit with endodontic need. In order to intervene in this cause-consequence cycle, parents and pregnant women should be motivated to schedule the first dental visit for their children within the first six months of life, with regular subsequent dental appointments, aiming to prevent the higher need for multiple endodontic treatments in the future.

Our findings also indicated that most of the patients (74%) presented with a minimum of 23 teeth, and more than 70% had at least one previous experience of endodontic treatment. Worth mentioning, having one previous RCT performed in the past resulted in a 67% less prevalence for needing multiple new endodontic treatment indications (*p*=0.023). Here, one may suggest that the prior experience of RCT in a single tooth may help the patient to pay more attention to his/her oral health and tooth pain condition, accounting positively to reduce the need for new endodontic therapy. However, this pattern was only associated with the presence of one previous RCT. This may be associated with patients who postponed oral health care and when it occurred, there was a higher number of teeth requiring endodontic treatment, reinforcing the importance of regular visits to dental office to prevent the demand for extensive and invasive dental interventions. Despite the fact that most of the patients have previously experienced endodontic treatment, no statistical association was found between the number of remaining teeth and the need for endodontic treatment, and no similar literature investigated this association previously, warranting more studies to confirm this finding.

Among all the teeth requiring RCT, most (73.9%) encompassed maxillary teeth, especially premolars (44.9%) and incisors or canines (42.5%), and females were more significantly affected than males (*p*=0.016). Premolars were 64% less associated with the need for multiple RCT when compared to patients indicated for RCT in incisors or canines (*p*=0.020). The higher association between anterior teeth and the need for multiple RCT may be related to a poor oral hygiene of those patients. Indeed, despite incisors and canines being easier-to-clean, once they have more extensive flat surfaces as compared with posterior teeth [29], patients having poor oral hygiene even at the anterior position would face more frequently caries development, perhaps contributing to the increased indication for multiple RCT. More importantly, anterior teeth might undergo greater level of traumatic injuries and wear promoted by parafunctional habits, so that multiple teeth can be harmed [30, 31]. Meanwhile, upper (24%) and lower (16.2%) molars were the most frequently treated teeth in Germans and Argentines, respectively [14, 32]. Likewise, studies conducted in southern Brazilian cities revealed that molars accounted for the highest demand of endodontic treatment [19, 33]. No less important, another feasible explanation for the greater prevalence of anterior teeth requiring RCT in this study may relate to the locations of the collected data (Dental Schools), which are mainly composed by undergraduate students who tend to treat anterior teeth or premolars (*i.e*., commonly single-rooted teeth) during RCT training. Hence, this might be considered a limitation of this study, since the literature clearly advocates that dental caries affect more frequently permanent molars [34].

Most Brazilians (70%) rely exclusively on public health care, including dental procedures, so this high demand of patients may prolong waiting time to begin treatment [35]. Public Dental Schools in Brazil are included in this unique health system (SUS), offering free RCT treatment to the population. Despite the usual long waiting time for dental procedures offered by the SUS, in the current study, 68.6% of the patients waited only 15 days to receive the treatment, which differs from earlier data reported in São Paulo, where patients used to wait 33 days at least to be treated [3]. Longer periods of waiting time were also described by services offered in Rio Grande do Sul and Minas Gerais (i.e., two Federal States in Brazil, in south and southeast areas, respectively), in which the patients had to wait for 90 days and 5 months in total to receive RCT [5, 36, 37]. Reduced waiting time appears to be a reflex of a simultaneous system performed in Dental Schools, since several students perform RCT at the same time under supervision of Professors, reaching a higher number of patients. Also, the efficient internal organization in scheduling patients reflects a faster service scenario. However, there is a dearth of studies comparing the impact of waiting time in Dental Schools to other public or private services.

Pulp and periapical status of teeth in this study demonstrated that 62.8% received pulp necrosis diagnosis without periapical involvement (44.4%). Our data resembles a previous survey in Sweden (38.1% of pulp necrosis) [9]. Conversely, it diverges from studies conducted in some countries in South America, which described irreversible pulpitis and acute apical periodontitis as the most prevalent diagnoses [33, 38, 39]. A recently published study also advocated that half of the world population have at least one tooth diagnosed with apical periodontitis [40]. To achieve the endodontic diagnosis, 61.4% of the cases relied on patient report, which was significantly more relevant within female patients (*p*=0.014). Also, women were significantly more affected by pain symptomatology than men (*p*=0.015). Apparently, this association may be explained by the easier communication of women during their report as well as due to the higher prevalence of oral pain in females caused by hormonal variations [41, 42]. To our knowledge, this is the first study to associate the female report with the accurate diagnosis in endodontics. Even though previous studies described pain as the main cause for patients seeking endodontic treatment in an urgency dental service (52.6%–70%) [33, 39], in the current study, most of the patients (42.5%) did not report pain at the first clinical appointment. A possible explanation for this dissimilarity leans on the current dental service where patients frequently had previously received pain relief in an urgency service and/or had been formerly selected.

In this study, most of the teeth had been previously restored (45.4%) at the time of RCT, and they were also significantly more prevalent in females (*p*=0.018). The most common types of restorative material recorded were temporary material (48.2%) and composite resin (37.5%), extending to one or two surfaces of the teeth (76%). Despite the low prevalence of amalgam restorations (12.5%), these patients exhibited 289% more prevalence of multiple needs for endodontic treatment when compared to teeth temporarily restored (*p*=0.077). This almost significant association may be explained since old amalgam restorations might have suffered from wear, fractures, and/or microleakage over time, endangering pulp vitality. A recent study exhibited the presence of restorations as the predominant feature in teeth requiring RCT (83.6%), supporting the harm of inadequate coronal sealing to pulp tissue, as well as the unlikely solely causative association between RCT and dental caries [9]. This fact corroborates with our findings that demonstrated high prevalence of inadequate restorations as a predisposing factor of RCT need, emerging further research to test this assumption. Studies demonstrated that composite resin restorations possess low failure rates (1%–5%) at a 5-year-period follow-up [43]; however, these data have exhibited increasing values along ten years (2006 to 2016), which was mainly caused by fracture of restorative material (39.3%) or due to secondary caries (25.8%) [44]. Preventive strategies investigating the reasons whether patients do not receive definitive restorations and taking into consideration the quality of materials and restorative techniques used both at public and private dental services emphasize the importance of periodic dental visits, aiding with the detection of microleakage in functioning restorations.

Interestingly, not only did decayed or restored teeth require RCT in our study, but also sound teeth exhibited greater prevalence of multiple RCT needs. Here, the majority of sound teeth presented wear caused by bruxism, which may frequently encompass large dental structure loss in multiple teeth. Ultimately, dental wear can cause dentinal sensibility due to the proximity to pulp tissue, leading to the multiple needs for RCT and prosthetic rehabilitation to reestablish patient vertical dimension. Despite the lack of statistical associations between bruxism and the need for multiple RCT, further studies should investigate this aspect, since the number of patients developing parafunctional habits due to stressful routine is increasing [45].

The present findings have also registered that most of the teeth had more than two-thirds of sound surfaces (62.3%), and the restored or decayed surfaces did not advance into the subgingival region in 85.5% of the cases. Thus, the teeth could be restored with direct restorations (78.3%). This finding contrasts with a report from Sweden which found 83.5% of teeth exhibiting significant restored surfaces with tooth substance loss of more than a third of the dental crown (71.3%) [9]. Collectively, these data may contribute to awareness of oral health authorities to improve the quality of restorative materials and provide professional capacitation according to the main demand in this population. The present study was conducted with a limited sample, which included all the patients referred to endodontic treatment in two public Dental Schools over one-year period. Large-scale validation of the current findings is essential. Future studies should assess data from patients of other dental facilities and compare collected data with the risk of caries for each patient.

In conclusion, this study described the main factors related to endodontic treatment indications that may help clinicians understand the conditions of tooth receiving RCT, guiding them in the treatment plan from the clinical exams to the final restorations. Our findings can support local authorities to invest in preventive strategies, high-quality restorative materials, and, last but not least, professional education, targeting offering a better treatment for patients in public services. Furthermore, clinicians should guide their patients concerning the importance of periodic dental visits and appropriated oral hygiene, which are usually neglected in the stressful routine of young adults. This could perhaps decrease the number of RCT indications mainly in the most affected groups verified in this study.

## Figures and Tables

**Table 1:** Bivariate analysis associating gender with patient-/tooth-related conditions for RCT indication (*n* = 207) (Chi-square test; *p* < 0.05).

Patient-related conditions	Total *n* (%)	Men *n* (%)	Women *n* (%)	*p*
Age				
18 to 39 years	144 (69.6)	50 (24.2)	94 (45.4)	0.997
40 to 59 years	49 (23.7)	17 (8.2)	32 (15.5)	
≥60 years	14 (6.8)	5 (2.4)	9 (4.3)	
Age at the first dental visit				
<7 years	26 (12.6)	9 (4.3)	17 (8.2)	0.990
7 to 20 years	163 (78.7)	57 (27.5)	106 (51.2)	
>20 years	18 (8.7)	6 (2.9)	12 (5.8)	
Frequency of dental hygiene				
≥3 times	121 (58.5)	39 (18.8)	82 (39.6)	0.361
≤2 times	86 (41.5)	33 (15.9)	53 (25.6)	
Number of permanent teeth				
≥29 teeth	57 (27.5)	27 (13.0)	30 (14.5)	0.067
23 to 28 teeth	96 (46.4)	26 (12.6)	70 (33.8)	
17 to 22 teeth	36 (17.4)	14 (6.8)	22 (10.6)	
≤16 teeth	18 (8.7)	5 (2.4)	13 (6.3)	
Number of teeth with previous RCT				
None	51 (24.6)	27 (13.0)	24 (11.6)	0.005
1 tooth	108 (52.2)	28 (13.5)	80 (38.6)	
2 to 3 teeth	37 (17.9)	15 (7.2)	22 (10.6)	
≥4 teeth	11 (5.3)	2 (1.0)	9 (4.3)	
Number of teeth indicated for RCT				
1 tooth	166 (80.2)	54 (26.1)	112 (54.1)	0.570
2 teeth	30 (14.5)	13 (6.3)	17 (8.2)	
3 teeth	6 (2.9)	3 (1.4)	3 (1.4)	
4 teeth	5 (2.4)	2 (1.0)	3 (1.4)	

Tooth-related conditions				

Tooth position				
Mandible	54 (26.1)	26 (12.6)	28 (13.5)	0.016
Maxilla	153 (73.9)	46 (22.2)	107 (51.7)	
Tooth group				
Incisor/canine	88 (42.5)	36 (17.4)	52 (25.1)	0.097
Premolar	93 (44.9)	25 (12.1)	68 (32.9)	
Molar	26 (12.6)	11 (5.3)	15 (7.2)	
Waiting time for RCT				
Up to 15 days	142 (68.6)	51 (24.6)	91 (44.0)	0.877
15 to 30 days	19 (9.2)	6 (2.9)	13 (6.3)	
≥30 days	46 (22.2)	15 (7.2)	31 (15.0)	
Pulpal diagnosis				
Normal pulp	9 (4.3)	3 (1.4)	6 (2.9)	0.702
Necrosis	130 (62.8)	49 (23.7)	81 (39.1)	
Asymptomatic irreversible pulpitis	29 (14.0)	9 (4.3)	20 (9.7)	
Symptomatic irreversible pulpitis	39 (18.8)	11 (5.3)	28 (13.5)	
Periapical diagnosis				
PAI 1	92 (44.4)	30 (14.5)	62 (30.0)	0.392
PAI 2 and 3	71 (34.3)	29 (14.0)	42 (20.3)	
PAI 4 and 5	44 (21.3)	13 (6.3)	31 (15.0)	
Consideration of patient's report during diagnosis^a^				
No	80 (38.6)	36 (17.4)	44 (21.3)	0.014
Yes	127 (61.4)	36 (17.4)	91 (44.0)	
Symptomatic at the clinical appointment				
No	123 (59.4)	51 (24.6)	72 (34.8)	0.015
Yes	84 (40.6)	21 (10.1)	63 (30.4)	
Pain duration before RCT				
Without pain	88 (42.5)	32 (15.5)	56 (27.1)	0.907
Up to 15 days	24 (11.6)	7 (3.4)	17 (8.2)	
16 to 30 days	31 (15.0)	10 (4.8)	21 (10.1)	
>30 days	64 (30.9)	23 (11.1)	41 (19.8)	
Dental crown condition				
Healthy	10 (4.8)	7 (9.7)	3 (2.2)	0.018
Satisfactory restoration	12 (5.8)	4 (5.6)	8 (5.9)	
Unsatisfactory restoration	94 (45.4)	25 (34.7)	69 (51.1)	
Dental caries	71 (34.3)	25 (34.7)	46 (34.1)	
Crown fracture or wear caused by bruxism	20 (9.7)	11 (15.3)	9 (6.7)	
Type of restorative material^b^				
Temporary material	54 (48.2)	14 (12.5)	40 (35.7)	0.260
Porcelain/ceramic	2 (1.8)	0 (0)	2 (1.8)	
Composite resin	42 (37.5)	12 (10.7)	30 (26.8)	
Amalgam	14 (12.5)	7 (6.3)	7 (6.3)	
Number of restored surfaces^b^				
1 face	36 (32.1)	11 (9.8)	25 (22.3)	0.936
2 faces	49 (43.8)	15 (13.4)	34 (30.4)	
3 faces	21 (18.8)	5 (4.5)	16 (14.3)	
≥4 faces	6 (5.4)	2 (1.8)	4 (3.6)	
Tooth substance loss				
None	21 (10.1)	8 (3.9)	13 (6.3)	0.956
≤1/3	129 (62.3)	45 (21.7)	84 (40.6)	
1/2	40 (19.3)	14 (6.8)	26 (12.6)	
≥2/3	17 (8.2)	5 (2.4)	12 (5.8)	
Indication for periodontal surgery				
No	177 (85.5)	66 (31.9)	111 (53.6)	0.066
Yes	30 (14.5)	6 (2.9)	24 (11.6)	
Type of restoration after RCT				
Direct	162 (78.3)	60 (29.0)	102 (49.3)	0.196
Indirect	45 (21.7)	12 (5.8)	33 (15.9)	

RCT, root canal treatment; PAI, periapical index score. ^a^Sum of both patient's report during the anamnesis and clinical tests (e.g., cold sensibility test, tenderness to percussion and palpation). ^b^Statistical analysis was performed using 112 teeth, only including cases presenting some restorative material.

**Table 2:** Prevalence ratio (PR) of tooth- and patient-related factors associated with indication for RCT with (*∗∗*) and without (*∗*) adjustments in 95% of confidence intervals (95% CI) using Poisson regression analysis.

Independent variables	PR (95% CI)*∗*	*p*	PR (95% CI)*∗∗*	*p*
Gender				
Male	1			
Female	0.68 (0.40–1.18)	0.169		
Age				
18 to 39 years	1		1	
40 to 59 years	0.49 (0.20–1.19)	0.116	0.91 (0.24–3.37)	0.883
≥60 years	2.06 (1.04–4.08)	0.039	0.88 (0.26–2.95)	0.832
Age at the first dental visit				
<7 years	1		1	
7 to 20 years	0.96 (0.41–2.24)	0.920	0.65 (0.23–1.84)	0.419
>20 years	1.73 (0.62–4.82)	0.292	5.03 (1.15–22.0)	0.032
Frequency of dental hygiene				
≥3 times	1			
≤2 times	1.34 (0.78–2.31)	0.294		
Number of permanent teeth				
≥29 teeth	1			
23 to 28 teeth	1.08 (0.56–2.09)	0.820		
17 to 22 teeth	0.72 (0.27–1.90)	0.507		
≤16 teeth	1.44 (0.58–3.59)	0.435		
Number of teeth with previous RCT				
None	1		1	
1 tooth	0.54 (0.29–0.98)	0.044	0.33 (0.13–0.83)	0.018
2 to 3 teeth	0.64 (0.29–1.42)	0.274	0.43 (0.07–2.45)	0.338
≥4 teeth	0.62 (0.17–2.32)	0.476	1.38 (0.27–7.07)	0.697
Tooth position				
Mandible	1			
Maxilla	1.09 (0.58–2.08)	0.784		
Tooth group				
Incisor/canine	1		1	
Premolar	0.46 (0.25–0.83)	0.009	0.36 (0.15–0.85)	0.020
Molar	0.13 (0.02–0.88)	0.037	0.20 (0.03–1.61)	0.130
Waiting time for RCT				
Up to 15 days	1			
15 to 30 days	0.58 (0.15–2.23)	0.424		
≥30 days	1.54 (0.87–2.75)	0.140		
Pulpal diagnosis^a^				
Necrosis	1			
Asymptomatic pulpitis^b^	0.96 (0.44–2.11)	0.920		
Symptomatic pulpitis^b^	0.48 (0.18–1.28)	0.140		
Periapical diagnosis				
PAI 1	1			
PAI 2 and 3	1.22 (0.66–2.24)	0.523		
PAI 4 and 5	0.98 (0.46–2.10)	0.967		
Consideration of patient's report during diagnosis^c^				
No	1			
Yes	0.54 (0.32–0.94)	0.029		
Symptomatic at the clinical appointment				
No	1			
Yes	0.85 (0.48–1.50)	0.563		
Pain duration before RCT				
Without pain	1			
Up to 15 days	0.97 (0.40–2.32)	0.936		
16 to 30 days	1.05 (0.49–2.25)	0.908		
>30 days	0.72 (0.36–1.45)	0.362		
Dental crown condition				
Healthy	1		1	
Satisfactory restoration	0.33 (0.08–1.36)	0.126	0.39 (0.22–1.03)	0.097
Unsatisfactory restoration	0.32 (0.15–0.69)	0.004	0.25 (0.07–0.74)	0.001
Dental caries	0.31 (0.14–0.71)	0.005	0.18 (0.04–0.44)	0.001
Crown fracture or wear caused by bruxism	0.80 (0.35–1.82)	0.594	0.89 (0.15–6.42)	0.180

Type of restorative material^d^				
Temporary material	1		1	
Composite resin	2.36 (0.95–5.85)	0.065	2.00 (0.69–5.81)	0.204
Amalgam	1.29 (0.29–5.70)	0.741	3.89 (0.80–19.0)	0.093
Number of restored surfaces^d^				
1 face	1			
2 faces	0.46 (0.16–1.29)	0.139		
≥3 faces	1.08 (0.43–2.73)	0.871		
Tooth substance loss				
None	1			
≤1/3	0.86 (0.33–2.24)	0.750		
1/2	1.31 (0.47–3.69)	0.606		
≥2/3	1.85 (0.62–5.52)	0.268		
Indication for periodontal surgery				
No	1			
Yes	1.43 (0.73–2.79)	0.294		
Type of restoration after RCT				
Direct	1			
Indirect	1.16 (0.62–2.18)	0.643		

RCT, root canal treatment; PAI, periapical index score. ^a^Statistical analysis was performed using 198 teeth, only including cases presenting some pulpal alteration (necrosis or pulpitis). ^b^Irreversible pulpitis diagnosis. ^c^Sum of both patient's report during the anamnesis and clinical tests (e.g., cold sensibility test, tenderness to percussion and palpation). ^d^Statistical analysis was performed using 112 teeth, only including cases presenting some restorative material.

## Data Availability

The quantitative data used to support the findings of this study are included within the article.
